# Measuring the positive psychological well-being of people with rheumatoid arthritis: a cross-sectional validation of the subjective vitality scale

**DOI:** 10.1186/s13075-015-0827-7

**Published:** 2015-11-05

**Authors:** Peter C. Rouse, Jet J. J. C. S. Veldhuijzen Van Zanten, Nikos Ntoumanis, George S. Metsios, Chen-an Yu, George D. Kitas, Joan L. Duda

**Affiliations:** School of Sport, Exercise and Rehabilitation Sciences, University of Birmingham, Birmingham, UK; Department for Health, University of Bath, Bath, BA2 7AY UK; Department of Rheumatology, Dudley Group of Hospitals NHS Trust, Russells Hall Hospital, Dudley, West Midlands UK; Department of Physical Activity, Exercise and Health, University of Wolverhampton, Walsall, West Midlands UK; Health Psychology & Behavioural Medicine Research Group, School of Psychology & Speech Pathology, Curtin University, Perth, Western Australia

**Keywords:** Rheumatoid arthritis, Psychological well-being, Subjective vitality, Outcome measure

## Abstract

**Introduction:**

People with rheumatoid arthritis (RA) frequently suffer from compromised physical and psychological health, however, little is known about positive indicators of health, due to a lack of validated outcome measures. This study aims to validate a clinically relevant outcome measure of positive psychological well-being for people with RA. The first study examined the reliability and factorial validity of the Subjective Vitality Scale (SVS), whilst study 2 tested the instruments convergent validity.

**Methods:**

In study 1, National Rheumatoid Arthritis Society members (N = 333; *M* age *=* 59.82 years *SD* = 11.00) completed a postal questionnaire. For study 2, participants (N = 106; *M* age = 56 years, *SD* = 12 years) were those recruited to a randomized control trial comparing two physical activity interventions who completed a range of health-related questionnaires.

**Results:**

The SVS had a high level of internal consistency (α = .93, Rho = .92). Confirmatory factor analysis supported the uni-dimensional factor structure of the questionnaire among RA patients [χ = 1327 (10), CFI = 1.0, SRMSR = .01 and RMSEA = .00 (.00 - .08)]. Support for the scales convergent validity was revealed by significant (p < .05) relationships, in expected directions, with health related quality of life (*r* = .59), physical function (*r* = .58), feelings of fatigue (*r* = −.70), anxiety (*r* = −.57) and depression (*r* = −.73).

**Conclusions:**

Results from two studies have provided support for the internal consistency, factorial structure and convergent validity of the Subjective Vitality Scale. Researchers and healthcare providers may employ this clinically relevant, freely available and brief assessment with the confidence that it is a valid and reliable measure of positive psychological well-being for RA patients.

**Trial registration:**

ClinicalTrials.gov ISRCTN04121489. Registered 5 September 2012.

## Introduction

Rheumatoid arthritis (RA) is the most prevalent chronic inflammatory arthritis, characterized by periods of remission and flares in disease activity unique to each individual. Untreated, poorly controlled or active RA leads to joint pain, stiffness, and swelling with eventual structural damage in affected joints [1]. In addition to these symptoms, RA patients commonly experience life dissatisfaction and psychological distress. However, less is known about positive indicators of well-being and positive functioning in RA patients, partly due to a lack of validated psychometric instruments. This study aims to fill this gap and provide evidence regarding the validity and reliability of the Subjective Vitality Scale (SVS) as a measure of positive psychological well-being for use with RA patients.

### Measuring psychological well-being

The psychological well-being of patients with RA is frequently compromised [2] due to fluctuations in disease activity; consequently, a body of research exists that investigates the physical concomitants of mental health and physical function in this clinical population. The mental health and psychological functioning of RA patients has most frequently been operationalized through measures of depression, anxiety and quality of life [2–5]. Research emphasis on anxiety and depression is not surprising given the increased prevalence in RA patients [6]. Over 25 years ago, Anderson and colleagues pointed to the high levels of depression and anxiety in RA patients [7]. Therefore, it is clear that anxiety and depression provide an important indicator of the psychological distress that RA patients experience.

However, psychological well-being does not simply refer to an absence of negative pathologies, such as anxiety and depression [8]. It is also important to consider the degree to which positive psychological states are experienced. Fewer studies have investigated the characteristics and correlates of positive psychological well-being in RA [9]. Quality of life is frequently employed as a measure of psychological well-being in patients with RA [10]. For example, Treharne and colleagues [9] investigated the effects of disease duration and psychosocial factors (i.e., social support) on quality of life, assessed using the Quality of Life Scale [11]. The Quality of Life Scale was developed and validated for use among rheumatic patients, and measures participants’ subjective satisfaction with their quality of life in 16 areas of importance (e.g., financial security and health). Treharne and colleagues [9] revealed that quality of life was highest in patients who had greater levels of optimism, more social support and in those with positive perceptions of the consequences of RA.

The Short Form 36 Health Survey (SF-36) [12] has also been employed as a measure of quality of life in RA patients [13, 14]. The SF-36 captures a variety of components of positive functioning, including physical function, body pain, vitality, role limitations- physical, role limitations- emotional general health, social function, and mental health. Measures of positive and negative affect have also been used as an assessment of psychological well-being in RA patients [2, 15, 16]. Coty and Wallston [2] showed that women with RA had lower levels of positive affect, compared to healthy women.

Although measures of quality of life and positive affect can be considered positive indicators of well-being, contemporary conceptualizations suggest that these facets center on more hedonic aspects of well-being (i.e., pleasure or happiness) and fail to adequately capture eudaimonic well-being [8]. When individuals experience eudaimonia, they experience optimal functioning. For example, successful treatment of the physical symptoms of RA may help to increase quality of life and have people in a better mood (e.g., stemming from their enhanced physical function) but the impact of the treatment on one’s overall psychological health and functioning may not be optimized [10]. Therefore, the vitality subscale of the SF-36 is grounded in a measure of hedonic well-being. In contrast, the Subjective Vitality Scale was specifically developed as a measure of eudaimonic well-being and explicitly captures qualities such as energy and spirit.

### Subjective vitality

Subjective vitality is an indicator of eudaimonic well-being that appears particularly salient for RA patients. Conceptualized as a specific and subjective positive psychological state, subjective vitality has been defined as a sense of feeling alive, vital, and full of energy [17]. This definition of subjective vitality aligns with the World Health Organization’s definition of psychological well-being as ‘a state of well-being in which every individual realizes his or her own potential, can cope with the normal stress of life, and can work productively and effectively’ [18]. Ryan and Frederick [17] further defined subjective vitality as energy that is perceived to emanate from the self, with an internal locus of causality and is influenced by both psychological and physical factors. It is an individual’s own appraisal of those factors that determine the degree of energy and spirit felt. For example, pain caused by RA, would interfere with one’s feelings of energy; however, the perceived meaning behind the physical symptom will determine the strength of vitality experienced. Therefore, subjective vitality appears to be a relevant measure of subjectively experienced positive psychological well-being that could provide additional information regarding the influence of RA on an individual’s overall physical and psychological functioning. Validating the SVS [17] could clarify the contribution of psychological well-being to the buffering role in coping with stress, the positive effect of interventions on disease course, and serve as an indicator of those vulnerable to depression [19].

The SVS was developed by Ryan and Frederick [17] in response to the lack of scientific investigation into optimal (in contrast to compromised) psychological functioning and has been validated and used as a measure of psychological well-being in a range of populations, including adult smokers, athletes, dancers, and university students [20–22]. However, to our knowledge no single scale assessing reported vitality (such as the SVS) has been validated for use with RA patients. The SVS was also selected for validation as it is freely available to use without payment or the need to request permission, increasing access and availability.

### Study aims

In order to ascertain whether the SVS is valid for use with RA patients, it is necessary to establish that all the items measure the same construct and that it is measuring what it is intended to measure, a state of positive psychological well-being. Study 1, therefore, tests the internal consistency of the scale by examining the Cronbach alphas and factorial validity through structural equation modeling. Study 2 assesses the construct validity of the SVS by testing the scale’s convergent validity. It is hypothesized that scores on the subjective vitality scale will demonstrate a positive correlation with other indicators of positive well-being and a negative relationship with indicators of compromised well-being/ill-being. Therefore, Study 2 examines the correlations between scores on the SVS with measures of physical function, quality of life, depression, anxiety, and fatigue.

## Methods

### Study 1 Methods

Members of the National Rheumatoid Arthritis Society (UK) were sent a pack containing an information sheet, consent form, questionnaire and a pre-paid stamped addressed envelope. Three hundred and thirty three (76 men and 256 women) participants returned the questionnaire and written informed consent forms (66 % return rate). Participants were middle aged (average age *=* 59.82 years *SD* = 11.00), predominantly white (96 %), married (68 %) and female (77 %). All participants had been diagnosed with RA (average duration = 14.80 years, *SD* = 11.34). Ethical approval was obtained from the National Health Services, Birmingham, East, North and Solihull Research Ethics Committee (10/H1206/59) ethics committee.

### Measures

The five item version of the Subjective Vitality Scale (SVS) [17, 22] was employed as a positive indicator of mental health and well-being in patients with RA. Participants responded to how they felt over the last two weeks, using a scale anchored by 1 (not at all true) to 7 (*v*ery true). See Table 1 for all questionnaire items. The SVS has shown good internal consistency in past work in the general population with Cronbach alphas ranging from 0.84 - 0.86 [22].

**Table 1:** Overall model fit and factors loadings for the Subjective Vitality Scale in patients with rheumatoid arthritis

Subjective Vitality Scale items	Factor loadings
I feel alive and full of vitality	.93
I have energy and spirit.	.90
I look forward to each day	.68
I nearly always feel alert and awake	.84
I feel I have a lot of energy	.89
Model Fit	
χ	1327 (10)
S-B Chi-square	29.56 (5)
CFI	1.0
SRMSR	.01
RMSEA	.00 (.00 -.08)

*S-B* Satorra-Bentler, *CFI* Comparative fit index, *SRMSR* standardized root mean square residual, *RMSEA* root mean square error of approximation

### Data analyses

Cronbach alphas were used to establish the internal consistency of the scale. An alpha ≥ .70 was considered to meet acceptable consistency. Given the debate regarding the dependability of Cronbach alpha as an estimator of reliability [23], Raykov’s Rho index was also employed. Raykov’s Rho index [24] provides an alternative measure of reliability for unidimensional scales that was developed for latent variable modelling [23]. Confirmatory factor analysis, employing the software package EQS 6.1 was conducted to examine the single factor structure of the Subjective Vitality Scale. The comparative fit index (CFI), standardized root mean square residual (SRMSR) and root mean square error of approximation (RMSEA) were used to determine overall model fit. As proposed by Hu and Bentler [25], a model demonstrates good fit when the CFI is ≥ .95, the SRMR is ≤ .08 and the RMSEA is ≤ .06. Questionnaire items with loadings ≥ .4 are considered to be representative of the construct; however, loadings ≥ .63 are considered very good [26].

### Study 1 Results

Descriptive statistics revealed that the mean subjective vitality score was moderate to low (*M* = 3.65 SD = 1.49) with 7 being the highest score. The SVS revealed a good level of internal consistency (α = .93, Rho = .92) and Mardia’s normalized estimate of multivariate kurtosis was 7.75. Scores on Mardia’s coefficient that are greater than 7 were initially thought to represent data with a multivariate non-normal distribution [27]; however, Bentler [28] later suggested a more robust cut-off (>5). The multivariate distribution of the present data does not meet these recommendations indicating the multivariate distribution is non-normal. Therefore, the Satorra-Bentler (S-B) chi-square test will be employed as it is the most robust test statistic with data that are multivariate non-normal. The S-B chi-square test incorporates a scaling correction that considers the model, estimation method and kurtosis values, thereby producing a more trustworthy fit index [29]. Confirmatory factor analysis demonstrated a good fit to the single factor questionnaire in patients with RA (Satorra-Bentler chi-square = 29.56 (5), CFI = .98, SRMR = .03 and RMSEA = .13 CI = .08 - .17). The standardized loadings ranged from .68 - .93 (See Table 1 for all standardized loadings).

### Study 2 Methods

For Study 2, participants were enrolled in a randomized controlled trial [ISRCTN04121489] investigating the effects of physical activity on cardio-respiratory fitness [30]. Ethical approval was obtained from the National Health Services, Birmingham, East, North and Solihull Research Ethics Committee (10/H1206/59). Data were used from the baseline assessment point. All participants provided written informed consent prior to participating in the study. Participants (*N* = 106) were predominantly married (66 %), middle aged (*M* age = 54.5 years, *SD* = 12.3 years), and female (68 %), with only 31 % employed. On average, disease duration was 7.4 years (*SD* = 8.6 years).

### Measures

In addition to the Subjective Vitality Scale, the following measures were completed by study participants:

#### Physical function

The Anglicized version of the 40 item Stanford Health Assessment Questionnaire (HAQ) [31] was used to assess patients’ physical function. Participants rated their ability (over the past week) to carry out 20 activities within eight aspects of daily living (dressing/grooming, rising, eating, walking, hygiene, reach, grip, and errands/tasks) on a four point scale from ‘without any difficulty’ to ‘unable to do’. For each aspect participants also responded to whether they receive assistance from people or use specific devices. The HAQ has been shown to possess high levels of internal consistency (α = .94) in patients with RA [32].

#### Quality of life

The EuroQol [33] self-assesses health related quality of life across five domains: mobility, self-care, usual activities, pain, discomfort and depression/anxiety. Each item has three levels of severity: ‘no problems’, ‘some problems’ and ‘severe problems’. The EQ-5D has previously been employed as a measure of health related quality of life in patients with RA [34].

#### Anxiety and depression

The Hospital Anxiety and Depression Scale (HADS) [35] measures participants’ depressive and anxiety symptoms. The HADS consists of 14 items (seven each for anxiety and depression) rated on different four point scoring systems. The subscales are internally consistent (α = .85 and .78, respectively) and have demonstrated convergent validity in the case of RA patients [9].

#### Fatigue

The 16-item Multidimensional Assessment of Fatigue (MAF) measures four dimensions: severity, distress, degree of interference in activities of daily living, and timing. Participants are asked to rate to what degree fatigue interferes with fourteen items, such as household chores, on a scale from 1 “not at all” to 10 “a great deal”. Two additional items have multiple-choice responses [36]. Participants were asked to reflect on fatigue patterns for the past week.

## Results

RA patients recruited to an exercise intervention experienced feelings of vitality (mean 4.01 SD = 1.55), fatigue (25.94 SD = 11.96), quality of life (0.67 SD = 0.24), physical function (0.52 SD = 0.49), low levels of anxiety (6.85 SD = 4.28), and depression (4.85 SD = 3.47). Pearson correlation coefficients revealed that the SVS was significantly and positively related to health related quality of life and physical function. Conversely feelings of subjective vitality were significantly and negatively related to feelings of fatigue, anxiety, and depression (see Table 2).

**Table 2:** Pearson correlation coefficients between subjective vitality and measures of well-being in patients with rheumatoid arthritis from Study 2

Measures			Subjective Vitality
	Mean	SD	*r*
Subjective vitality	4.05	1.57	
Quality of life	.68	.24	.59
Physical function	.51	.48	.58
Global fatigue	26.0	11.79	-.70
Anxiety	6.78	4.24	-.53
Depression	4.82	3.48	-.73

## Discussion

Currently, a limited number of validated measures of positive psychological well-being exist for patients with rheumatoid arthritis (RA) contributing to a heavy reliance on negative indicators of well-being (such as anxiety and depression) in this clinical population. The purpose of this paper was to validate the SVS as a brief, freely available measure of positive psychological well-being in RA patients so that it can be used for both clinical and research purposes. The SVS appears to be a particularly relevant measure as it targets what is typically examined and counters the negative symptoms patients with RA experience (e.g., lack of energy).

### Construct validity

The aim of the current study was to assess the internal consistency and construct validity of the SVS for patients with RA. Study 1 provides support for the internal consistency of the SVS. High Cronbach alpha and Raykov’s rho scores demonstrate that the way patients with RA rated the five different items corresponded to each other. Similar to other populations, such as university students [22], patients with RA appear to have understood the meaning behind each item. This finding suggests that the amount of random error generated by the SVS in RA patients due to guessing or misunderstanding is low [22]. Study 1 also provided support for the factorial validity of the SVS through confirmatory factor analysis. Data collected from predominantly female middle aged members of NRAS demonstrated a good fit to the single factor structure of the questionnaire as recommended by the most commonly employed criteria proposed by Hu and Bentler [25]. However, it is noteworthy that although the CFI, Chi-square, and SRMSR highlight a good fit, the RMSEA failed to meet the recommended cut-off of ≤ .08. This inconsistency may be explained by the small number of variables (items) that are included in the CFA. The RMSEA is expressed per degree of freedom and is, therefore, sensitive to the number of estimated parameters in the model [29]. Kenny and McCoach [37] highlight that the RMSEA tends to improve as the number of the variables included within the model increases.

### Patient profile

Results from Study 1 indicated that RA patients scored moderate to low on feelings of subjective vitality. Mean scores from participants in Study 2 were slightly higher. The elevated score found in Study 2 could be explained by the fact that participants were RA patients who had signed up to participate in an exercise intervention. Therefore, we could surmise that participants from Study 2 felt that they possessed sufficient energy to engage in physical activity. It is noteworthy, however, that subjective vitality scores from both studies are suppressed compared to healthy populations. Vlachopoulos and Karavani [38] investigated perceptions of subjective vitality in 388 healthy exercisers between 18- and 61-years old and found a mean score of 4.96 (SD = 1.36). Due to a lack of previous research employing the SVS in RA, it is not possible to draw direct comparison with RA patients’ scores from other studies. However, some measures of health related quality of life include a vitality dimension (e.g., SF-36) and studies that have used these measures indicate that RA patients score just over midway on the scale whereas the general population scored higher [39]. Therefore, scores on the SVS employed in the present studies appear to replicate and support those generated from vitality subscales taken from measures of quality of life in RA patients.

### Convergent validity

Study 2 provided support for the convergent validity of the SVS through significant positive correlations with indicators of well-being and enhanced functioning. Higher scores on the SVS were associated with higher scores for health related quality of life, one of the most frequently employed measures of positive well-being in RA [10]. Therefore, if an RA patient feels full of energy and alive they are also more likely to perceive that they have a better quality of life. Participants who perceived themselves to be experiencing high levels of vitality were also more likely to report that they have better physical function. However, previous research suggests that physical impairment does not necessarily denote poor psychological well-being [10]. Ryan and Frederick [17] also indicate that it is the individual’s appraisal of the impairment that determines the consequence for feelings of subjective vitality. RA is characterized by periods of flares and remission; therefore, it would be important for future research to investigate whether feelings of subjective vitality can persist in the context of pain and decreased physical functioning caused by flares in disease activity.

Significant medium to strong negative relationships were revealed between the SVS and measures of compromised well-being/ill-being. A strong negative relationship was shown between SVS and perceptions of fatigue supporting the hypothesized inverse relationship between the positive and negative indicators of well-being. This finding replicates previous research in RA patients indicating that feelings of vitality (SF-36) are strongly and negatively associated with patients’ experience of fatigue [39, 40]. Medium to strong negative correlations with anxiety and depression were also revealed demonstrating that patients with RA who reported higher levels of subjective vitality report fewer indications of diminished mental health. The significant association observed between subjective vitality and depressive symptoms replicates and extends findings from previous research in other populations, such as adolescents [41]. Therefore, correlation analyses from Study 2 support the use of the SVS as a positive indicator of psychological well-being in patients with RA.

### Clinical relevance and future research

The SVS was selected for validation as it was systematically developed as a measure of eudaimonic well-being that can be meaningfully integrated into theories of human functioning [17]. In contrast, the other available measure is a subscale from the SF-36 that is designed to measure quality of life. Important philosophical differences in the conceptualization of well-being distinguish these two instruments and highlight the importance of validating the SVS for patients with RA. Inspection of the SVS also reveals that only items pertaining to feelings of energy and aliveness are included, whereas, the SF-36 includes items relating to fatigue and weakness that could be confounded by negative states, such as depression, fatigue and other health related symptoms [17].

The SVS can provide clinically relevant data on patients’ positive psychological well-being that is not currently gathered due to the lack of validated instruments. Reid and colleagues [42] highlight the utility and contribution of self-reports of subjective well-being in identifying individuals at risk of adverse health outcomes. Subjective well-being can also provide an additional criterion for examining the effectiveness of treatment programs. Quantitative data from the SVS would allow clinicians and rheumatology healthcare providers to track and assess the positive (rather than negative) psychological responses of RA patients to treatment. However, a psychometric instrument can only have clinical relevance if it can be feasibly administered, scored, and interpreted by the relevant parties. The SVS is a short five item questionnaire that RA patients would find easy to answer and complete with rheumatology healthcare providers able to score and interpret data quickly. Thus, we would suggest that the five item SVS is feasible to use in clinical practice. Future research can also employ the SVS to examine and test the effect of interventions, both behavioral and cognitive, on an indicator of positive psychological well-being in RA patients. The SVS can be used as a primary or secondary outcome with the confidence that it measures a positive indicator of well-being and optimal functioning for this population.

Despite the support provided in the present research for the use of the SVS with RA patients, it is important to highlight that this validation is somewhat limited by the predominantly female and middle aged sample that the data represent. However, RA is more prominent in women and most frequently but not always diagnosed in middle aged patients. The validity of the SVS may be further tested by examining whether the factor structure is invariant across gender and other RA characteristics, such as disease duration or severity.

## Conclusions

This study offers support for the internal consistency, construct and convergent validity of the SVS as a positive indicator of psychological well-being in patients with RA. Researchers and rheumatology healthcare providers may employ this clinically relevant, freely available and feasible instrument with the confidence that it is a valid measure of optimal function and well-being. Future research studies may also employ this measure to facilitate understanding of the potential mechanisms that underpin the positive psychological well-being in RA patients.

### Significance and innovations

- The mental health and psychological functioning of RA patients has most frequently been operationalized through measures of compromised health, such as depression and anxiety.
- Little is known about positive indicators of well-being and positive functioning in RA patients, partly due to a lack of validated outcome measures.
- Scores from the Subjective Vitality Scale correlated with indicators of both physical and psychological functioning in patients with RA.
- Researchers and rheumatology healthcare providers may employ the Subjective Vitality Scale as a clinically relevant and feasible outcome measure with the confidence that it is a valid measure of positive health and well-being.

## Abbreviations

RA: Rheumatoid arthritis
SVS: Subjective Vitality Scale
SF-36: Short Form Health Survey
HAQ: Health Assessment Questionnaire
MAF: Multiple Assessment of Fatigue
HADS: Hospital Anxiety and Depression Scale
CFI: comparative fit index
SRMSR: standardized root mean square residual
RMSEA: root mean square error of approximation
